# Higher Medical Education

**Published:** 1892-10

**Authors:** 


### HIGHER MEDICAL EDUCATION IN THE SOUTHERN STATES.

The circular recently sent out from Nashville, with the names of Drs. W. T. Briggs and W. D. Haggard, invites the medical schools of the South to a convention in Louisville, Ky., during the session of the Southern Surgical and Gynecological Association which will be held on the 15th, 16th and 17th of November, next.

The object of this conference is to adopt an uniform standard of requirements by all the Southern schools, and thus place them on an equal basis before the students who may seek a place for pursuing their studies. Without co-operation in the requirements for graduation, it is evident that nothing can be accomplished in advancing the cause of medical education either by lengthening the term, defining the preliminary qualifications, or exacting a thorough knowledge of the different branches for receiving the degree of M. D. Should the schools of one State require a three years' course of study, while others decline to do so, the most probable result would be, that students who are anxious to get a degree by the shortest route, without reference to a high order of professional attainments, should patronize the schools having a two years' course. Had the proposed bill before the legislature of Georgia become a law and exacted from the schools of this State a requirement of three years' attendance on the part of the students, it must have proven suicidal to the Georgia medical colleges, while colleges of other Southern States continued to graduate students with only two years' attendance. The example of the medical college of South Carolina at Charleston in establishing a three years' course, serves to illustrate this point in a most signal manner. If the great falling off in numbers, which has followed this premature step, does not suffice to convince those in authority in Georgia of the fate which must have attended a requirement of three years in advance of the other schools in the South, no reasoning can avail to satisfy them.

However desirable it may be to raise the standard of medical education, the question of expediency in undertaking to advance the requirements without co-operation, stares us in the face; and if all can be induced to move together by such action as is proposed in this convention, the interests of all concerned will be promoted.

The Southern Medical College has signified its acceptance of the invitation to join in the convention at Louisville, and it is to be hoped that every Southern school may accede to the proposition to adopt a three years' course and insist upon proper preparation for entering upon the study of medicine.

Some definite understanding should also be reached in regard to the qualifications for graduation, so that a wholesale granting of diplomas shall discredit the school which tacitly, if not publicly, gives all applicants to understand that they will receive their degree.

It is not enough that the graduates of such a school shall fail to pass the examination of the medical boards in a neighboring State, however humiliating this may be to the poorly trained victim, but the school discharging such material upon the suffering people of this country should be held to accountability.

The faculty of the Southern Medical College have sought diligently to eliminate the chaff from the wheat in the final examination of students; and the small percentage of failures in the examinations before the Alabama Medical Board, in comparison with those of another school in Atlanta, gives assurance to the public of the faithful work done by the professors of the Southern Medical College in the past, and affords a guarantee of what may be expected in the future. With a commodious new building for the session which opens October 4, and valuable additions to the corps of instructors, it is expected that advantages will be presented to students, equal, if not superior, to those afforded by any medical college in the United States.

While some of the chairs have been vacated by the former incumbents, they will be ably filled by newly elected professors.

Dr. W. S. Elkin has been elected to the professorship of operative surgery, and his recognized ability gives him a just title to this position.

Dr. Floyd W. McRae, who is well and favorably known in this section, has spent much time in attending the polyclinics of New York, and is eminently fitted for his professorship of physiology.

Dr. Henry R. Harris completed his medical education in the Jefferson School of Philadelphia, and afterwards pursued a regular course of instruction in laboratory work, thus gaining knowledge which qualifies him fully for the chair of chemistry to which he has been elected.

Dr. Logan M. Crichton, having established a reputation in diseases of the nose and throat, fills all the requisites for instruction in this department to which he has been elected.

Dr. C. D. Roy, after serving as the assistant to the professor of diseases of the eye, ear, nose and throat, has been perfecting his studies in his specialty in Europe, so that he will meet satisfactorily the full measure of expectation for the chair of eye and ear diseases.

Dr. L B. Grandy, whose proficiency and skill has already been shown by his work, has been assigned the position of demonstrator of anatomy.

Nothing is requisite to be said in regard to the former members of the faculty of this institution, who still continue to discharge the duties of their several professorships, as they are known of all men to be competent and faithful in their work of instruction.

This reference to the Southern Medical College is intended to put all upon notice that its professors are determined to maintain the position of progressive medical teachers, and that they are now ready to move forward with the co-operation of the other Southern schools, in elevating the standard of medical education. J. McF. G.

